# The Roles of Stress and Pressure in Multicaloric Effects of Shape Memory Alloys: Mechanisms and Recent Advances

**DOI:** 10.3390/ma19214464

**Published:** 2026-10-23

**Authors:** Linyan Zhang, Changshuo Zhang

**Affiliations:** 1 Tianmushan Laboratory (Zhejiang Provincial Laboratory for Aviation), Hangzhou 311312, China; 2 International Innovation Institute, Beihang University, Hangzhou 311312, China; zb2557435@buaa.edu.cn

**Keywords:** shape memory alloys, multicaloric effects, uniaxial stress, hydrostatic pressure, multi-field coupling

## Abstract

Shape memory alloys (SMAs) undergo reversible martensitic transformations that generate caloric responses and offer potential for solid-state cooling. This review critically examines how uniaxial stress and hydrostatic pressure regulate the caloric and multicaloric behavior of SMAs, with particular emphasis on their coupling with magnetic fields. Uniaxial stress acts directionally through transformation strain and variant selection, whereas hydrostatic pressure modifies phase stability through the transformation volume change between austenite and martensite. Their combined or sequential application with magnetic fields is analyzed in terms of phase fraction, transformation path, hysteresis, reversibility, and operating-temperature range. To clarify the available evidence, direct field-driven caloric responses are distinguished from processing-induced changes in microstructure and internal stress that indirectly affect subsequent caloric behavior. Recent experimental and theoretical advances are compared using caloric amplitude, loading and unloading responses, energy dissipation, cyclic stability, and applied-field requirements. The results show that appropriately designed coupled-field protocols may enhance caloric responses and reduce hysteresis in selected systems; however, these effects depend on alloy composition, microstructure, field magnitude, temperature, and loading sequence. Standardized measurements and coupled thermodynamic-kinetic models are therefore needed to support the development of reversible and durable multicaloric cooling systems under device-relevant fields.

## 1. Introduction

Solid-state refrigeration based on caloric effects has attracted considerable attention as an energy-efficient and environmentally friendly alternative to conventional vapor-compression technologies [1,2,3]. Among various caloric materials, shape memory alloys (SMAs), particularly NiTi and Ni-Mn-based Heusler systems, exhibit reversible martensitic transformations that give rise to pseudoelasticity and elastocaloric effects [1,4]. From a thermodynamic perspective, these transformations are highly sensitive to external mechanical stimuli, indicating a strong coupling between mechanical fields and phase stability.

In magnetic shape memory alloys (MSMAs), especially Ni-Mn-based systems, the coupling between magnetic and structural transitions gives rise to pronounced magnetocaloric and elastocaloric responses. These transformation behaviors can be effectively regulated by stress and hydrostatic pressure, which modify the free-energy landscape, transformation temperatures, and hysteresis. For example, combined stress and magnetic field enables large and reversible multicaloric responses in Ni-Co-Mn-Sn alloys [5], while hydrostatic pressure coupled with magnetic field effectively tunes martensitic transformation in Ni-Mn-based MSMAs [6], thereby modifying the entropy change, transformation hysteresis, and operating-temperature range.

Advances in additive manufacturing (AM), particularly laser powder bed fusion (LPBF), provide effective approaches for tailoring internal stress states and microstructures in SMAs. By enabling precise control of composition, defect structures, and residual stress distribution, AM techniques can regulate martensitic transformation behavior, including transformation temperature, hysteresis, and functional strain [7,8,9]. Furthermore, compositional tuning, such as Cu alloying, reduces transformation hysteresis and improves cyclic stability [10]. These processing strategies can modify internal stress states and transformation behavior and may therefore influence caloric performance indirectly.

Beyond processing strategies, multi-field coupling involving magnetic, electric, and mechanical stimuli further highlights the dominant role of stress and pressure in governing transformation behavior. External fields such as magnetic or electric stimuli can indirectly modulate internal stress states and alter transformation thermodynamics. For instance, electric-pulse-assisted processing has been reported to modify phase-transformation behavior, residual strain, microstructure, and superelastic properties in NiTi-based alloys [11,12,13]. However, because these studies did not directly determine caloric quantities such as entropy change or adiabatic temperature change, the reported results should be regarded as processing-induced changes with potential, rather than demonstrated, relevance to caloric performance.

Substantial progress has also been made toward more reversible and cyclically stable cooling. Current strategies include improving elastocaloric stability through precipitation and microstructural control, enhancing barocaloric responses through composition design, and regulating phase stability, hysteresis, and reversibility through coupled magnetic and mechanical fields [14,15]. These developments demonstrate that practical performance should be evaluated not only by peak caloric values but also by the required field magnitude, hysteresis, reversibility, and cyclic stability.

Recent reviews have examined elastocaloric and multicaloric technologies from complementary perspectives. Mañosa and Planes [16] discussed the thermodynamic foundations of elastocaloric effects and mechanically–magnetically coupled responses, whereas Sun and Liu [17] focused on magnetoelastic coupling and multicaloric effects in Ni-Mn-based Heusler SMAs. Li et al. [18] provided a broader overview of elastocaloric materials, manufacturing, simulation, and cooling devices, while other recent reviews further addressed material and microstructural optimization [19], AM of NiTi alloys [20], and elastocaloric system performance [21]. Nevertheless, the distinct roles of uniaxial stress and hydrostatic pressure have not been systematically compared as a central framework across SMA systems. Moreover, direct caloric responses under coupled fields have not been consistently distinguished from processing-induced changes with only indirect relevance to caloric performance, while quantitative comparisons of applied fields, caloric responses, hysteresis, and cyclic stability remain dispersed across the literature.

To address these gaps, this review systematically examines the distinct roles of uniaxial stress and hydrostatic pressure in the caloric and multicaloric responses of SMAs. Caloric responses induced by the simultaneous or sequential application of coupled external fields are evaluated separately from processing-induced changes that may only indirectly affect caloric performance. By integrating thermodynamic analysis, quantitative comparisons of applied fields and caloric metrics, and microstructure-transformation relationships, the review establishes a mechanistic framework linking phase stability, hysteresis, energy dissipation, reversibility, and cyclic stability.

## 2. Scope and Literature Search Methodology

This review focuses on experimental and theoretical studies of stress- and pressure-mediated caloric responses in SMAs, with particular emphasis on the coupling between mechanical and magnetic fields. Relevant publications were identified through targeted searches of the Web of Science Core Collection and Scopus, supplemented by reference tracing. The search combined “shape memory alloy” or “martensitic transformation” with terms related to caloric phenomena (“magnetocaloric,” “elastocaloric,” “barocaloric,” and “multicaloric”), external fields (“uniaxial stress,” “hydrostatic pressure,” “magnetic field,” and “coupled fields”), performance limitations (“hysteresis,” “cyclic stability,” and “functional fatigue”), processing (“laser powder bed fusion” and “electric-pulse treatment”), and modeling (“thermodynamic,” “phase-field,” and “constitutive modeling”). Relevant literature available up to September 2026 was considered. No lower publication-year limit was applied because earlier studies were required to establish the thermodynamic foundations and provide representative evidence for caloric and multicaloric responses. Particular attention was given to studies published between 2023 and 2026 to capture recent advances. Because this work is a critical narrative review rather than a systematic review or meta-analysis, the search aimed to identify representative studies within the defined scope rather than provide an exhaustive record of all publications.

Priority was given to peer-reviewed studies reporting: (i) caloric responses of SMAs under single, combined, or sequential stress, pressure, and magnetic fields; (ii) quantitative transformation or caloric data, including transformation temperature, entropy change, adiabatic temperature change, hysteresis, or cyclic stability; or (iii) processing-induced microstructural changes with identifiable relevance to subsequent caloric behavior. Selected non-peer-reviewed academic materials were used only as supplementary sources for established thermodynamic or methodological background and were not included in the quantitative comparison. Duplicate publications, studies outside the scope of SMAs, and publications lacking sufficient information on applied fields, processing conditions, or phase-transformation behavior were excluded from detailed discussion.

The selected studies were classified as direct caloric studies, processing-related studies, or theoretical and modeling studies. Direct caloric studies report entropy change, adiabatic temperature change, or exchanged heat, whereas processing-related studies were regarded as indirect evidence unless caloric quantities were measured after processing. Theoretical and modeling studies were considered separately to support the interpretation of field-dependent phase stability, transformation kinetics, microstructural evolution, and functional fatigue. Only values explicitly reported in the original publications were used for quantitative comparison; unreported quantities were marked as unavailable. Differences in composition, experimental protocol, field history, and measurement method were considered when comparing representative SMA systems.

## 3. Thermodynamic Basis and Evidence for Stress- and Pressure-Mediated Multicaloric Responses

### 3.1. Thermodynamic Framework and Determination of Caloric Quantities

The caloric responses of SMAs originate from entropy changes associated with field-driven martensitic transformations. Under a simplified scalar description, the differential of the molar Gibbs free energy for a system subjected to uniaxial stress (σ), hydrostatic pressure (*p*), and an external magnetic field (H) may be expressed in terms of the corresponding thermodynamically conjugate variables as [18,22,23]:

*dG* = −*SdT* − *Vεdσ* + *Vdp* − *md*(*μ*_0_*H*) (1)

where *S*, *V*, ε, and m denote the molar entropy, molar volume, uniaxial strain, and molar magnetic moment, respectively. Tensile stress and compressive hydrostatic pressure are taken as positive.

Defining ∆*X* = *X_M_* − *X_A_*, uniaxial stress, hydrostatic pressure, and magnetic field modify the relative stability of austenite and martensite through ∆ε, ∆*V*, and ∆*m*, respectively. Their application or removal produces an isothermal entropy change ∆*S* or an adiabatic temperature change ∆*T_ad_* [16,18,24]. Under multiple fields, the resulting response depends on the transformation fraction, field sequence, hysteresis, and cross-field interactions and therefore cannot generally be obtained by simply adding the corresponding single-field effects [5,6,24,25,26].

Uniaxial stress and hydrostatic pressure couple to martensitic transformations through different thermodynamic variables. Based on the phase-equilibrium condition and assuming a small variation in molar volume, their Clausius–Clapeyron relations can be expressed as [16,18,27]:

(2) $$\frac{dT_{t}}{\mathrm{d\sigma}}\approx -\frac{V\Delta \varepsilon }{\Delta S_{\mathrm{tr}}}$$

(3) $$\frac{dT_{t}}{\mathrm{dp}}=\frac{\Delta V}{\Delta S_{\mathrm{tr}}}$$

(4) $$\frac{dT_{t}}{\mathrm{dH}}=-\frac{\mu_{0}\Delta m}{\Delta S_{\mathrm{tr}}}$$

where *T_t_*, ∆*ε*, ∆*V*, ∆*S_tr_* and ∆*m* denote the equilibrium transformation temperature, transformation strain, molar-volume change, molar transformation-entropy change, and molar magnetic-moment difference, respectively. All phase differences are defined as ∆*X* = *X_M_* − *X_A_*.

These relations provide a direct basis for interpreting field-induced changes in transformation temperature and phase stability. Because the forward martensitic transformation in the SMA systems considered here generally has ∆*S_tr_* < 0, a positive transformation strain gives *dT_t_*/*dσ* > 0, indicating that tensile stress favors martensite with a positive strain along the loading direction. When martensite has a smaller molar volume than austenite, Δ*V* < 0 and *dT_t_*/*dp* > 0, so compressive hydrostatic pressure stabilizes martensite and raises *T_t_*. The magnetic-field dependence is governed by ∆*m*; when austenite has the larger molar magnetic moment, ∆*m* < 0, the magnetic field favors austenite and generally lowers *T_t_*. Thus, the signs and magnitudes of the transformation strain, molar-volume change, transformation-entropy change, and magnetic-moment difference determine the thermodynamic tendency of the applied fields to act cooperatively or competitively.

Uniaxial stress is directional and couples primarily to transformation strain, thereby influencing martensitic variant selection and producing an elastocaloric response [16,18,24]. In contrast, hydrostatic pressure is nominally isotropic and couples primarily to the volume discontinuity, producing a barocaloric response [16,24]. When martensite has a smaller molar volume than austenite, pressure generally stabilizes martensite and may increase the transformation temperature [6,28]. Therefore, uniaxial compression and hydrostatic pressure should not be treated as equivalent mechanical fields.

Recent barocaloric studies further demonstrate that pressure-driven caloric performance depends on the transformation-volume change, transformation-entropy change, pressure-induced transformation-temperature shift, and hysteresis. In polycrystalline Ni_54_Mn_23_Ga_23_, hydrostatic pressure modifies the martensitic transformation and barocaloric response, with a reported *dT_M_*/*dp* of approximately 15.89 K GPa^−1^, consistent with the positive slope predicted by Equation (3) for a lower-volume martensitic phase [29]. Descriptor-guided compositional design has also produced a giant barocaloric response in Ni-Cu-Mn-Sn alloys [30]. These results provide single-field reference points for assessing the effects of pressure-magnetic coupling discussed in Section 3.3.

Caloric effects can be evaluated directly by measuring the ∆*T_ad_*, latent heat, or heat exchanged during a field-driven cycle. Alternatively, the field-induced entropy changes may be estimated using Maxwell relations [22,24,27]:

(5) $$\Delta S_{\sigma }\approx \int_{\sigma_{1}}^{\sigma_{2}}V{\left(\frac{\partial \varepsilon }{\partial T}\right)}_{\sigma }\mathrm{d\sigma}$$

(6) $$\Delta S_{p}=-\int_{p_{1}}^{p_{2}}{\left(\frac{\partial V}{\partial T}\right)}_{P}\mathrm{dp}$$

(7) $$\Delta S_{H}=\mu_{0}\int_{H_{1}}^{H_{2}}{\left(\frac{\partial m}{\partial T}\right)}_{H}\mathrm{dH}$$

When the temperature and heat capacity vary only slightly during the field-induced process, the adiabatic temperature change may be approximated as [24,27]:

(8) $$\Delta T_{\mathrm{ad}}\approx -\frac{T}{C_{p}}\Delta S$$

where *C_p_* is the molar heat capacity. This approximation assumes an approximately reversible process and limited variation in *C_p_*.

Maxwell relations should be applied cautiously to first-order hysteretic transformations because they are derived for equilibrium state functions. Phase coexistence, metastability, incomplete transformation, and path-dependent initial states may lead to unreliable entropy estimates, including artificial entropy peaks [22,31]. Therefore, forward and reverse transformations should be evaluated separately using reproducible initial states, and indirect estimates should preferably be validated by calorimetry, Clausius–Clapeyron analysis, or direct ∆T_ad_ measurements.

**Figure 1:**
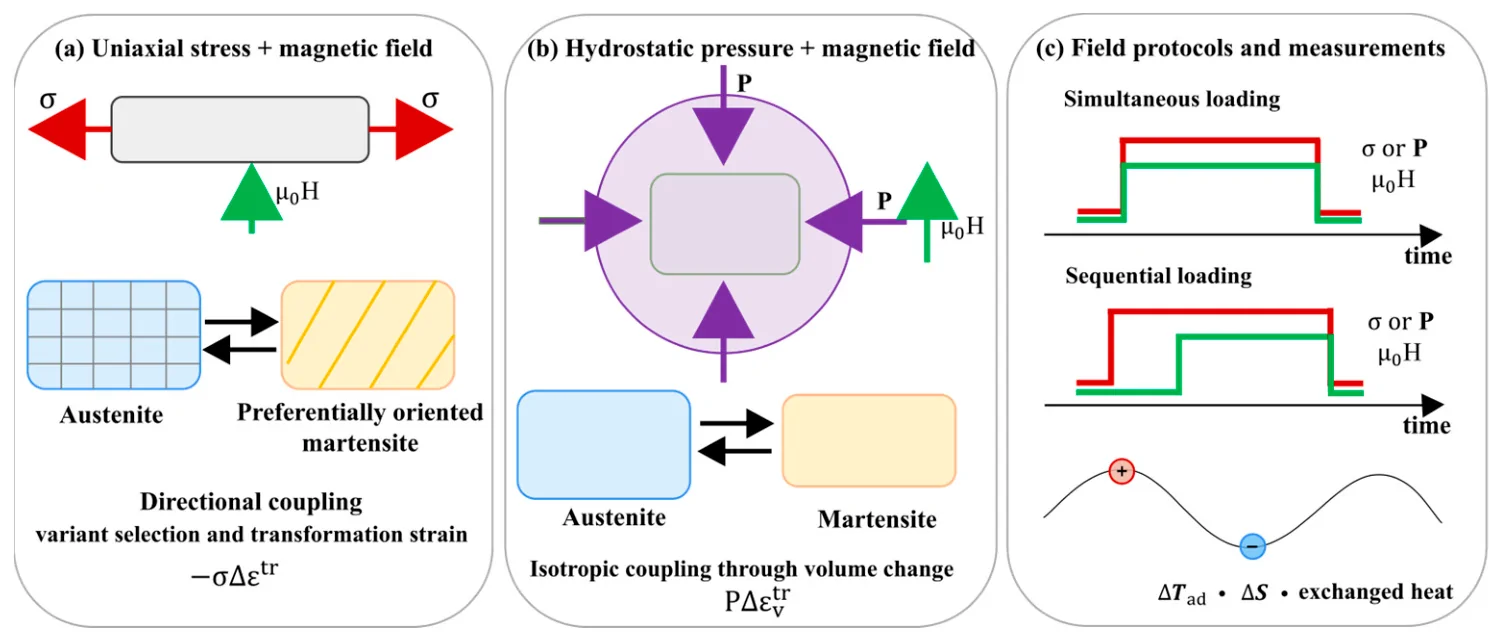
Schematic illustration of stress- and pressure-mediated multicaloric experiments in shape memory alloys: (**a**) coupled uniaxial stress and magnetic field, where directional stress acts through transformation strain and martensitic variant selection; (**b**) coupled hydrostatic pressure and magnetic field, where nominally isotropic pressure acts through the transformation volume change; and (**c**) representative simultaneous and sequential field-loading protocols and the associated caloric quantities, including ∆*T_ad_*, Δ*S*, and exchanged heat. Here, Δ*ε^tr^* and ${\Delta \varepsilon }_{v}^{\mathrm{tr}}$ represent the directional and volumetric transformation strains.

For coupled fields, independently measured single-field entropy changes cannot generally be added because the response depends on the transformation fraction, field sequence, hysteresis, and cross-field interactions. Accordingly, this review distinguishes directly measured caloric responses, indirect thermodynamic estimates, and processing-induced changes for which caloric quantities were not determined. Figure 1 schematically illustrates the distinct experimental configurations and coupling routes associated with uniaxial stress, hydrostatic pressure, and magnetic field.

### 3.2. Uniaxial Stress-Magnetic Field Coupling and Multicaloric Responses

Uniaxial stress couples directionally to transformation strain, thereby modifying phase stability and promoting martensitic variant selection or reorientation. Its effect depends on loading direction, crystallographic texture, grain constraints, and specimen geometry. Because variant accessibility and transformation strain can differ between tension and compression, the two loading modes may produce different critical stresses, hysteresis, recoverable strains, and caloric responses. Magnetic field couples instead to the magnetization difference between austenite and martensite. Their combined effect therefore depends on the relative stabilization of the two phases, variant selection, field magnitude, loading sequence, and transformation hysteresis [5,32].

**Figure 2:**
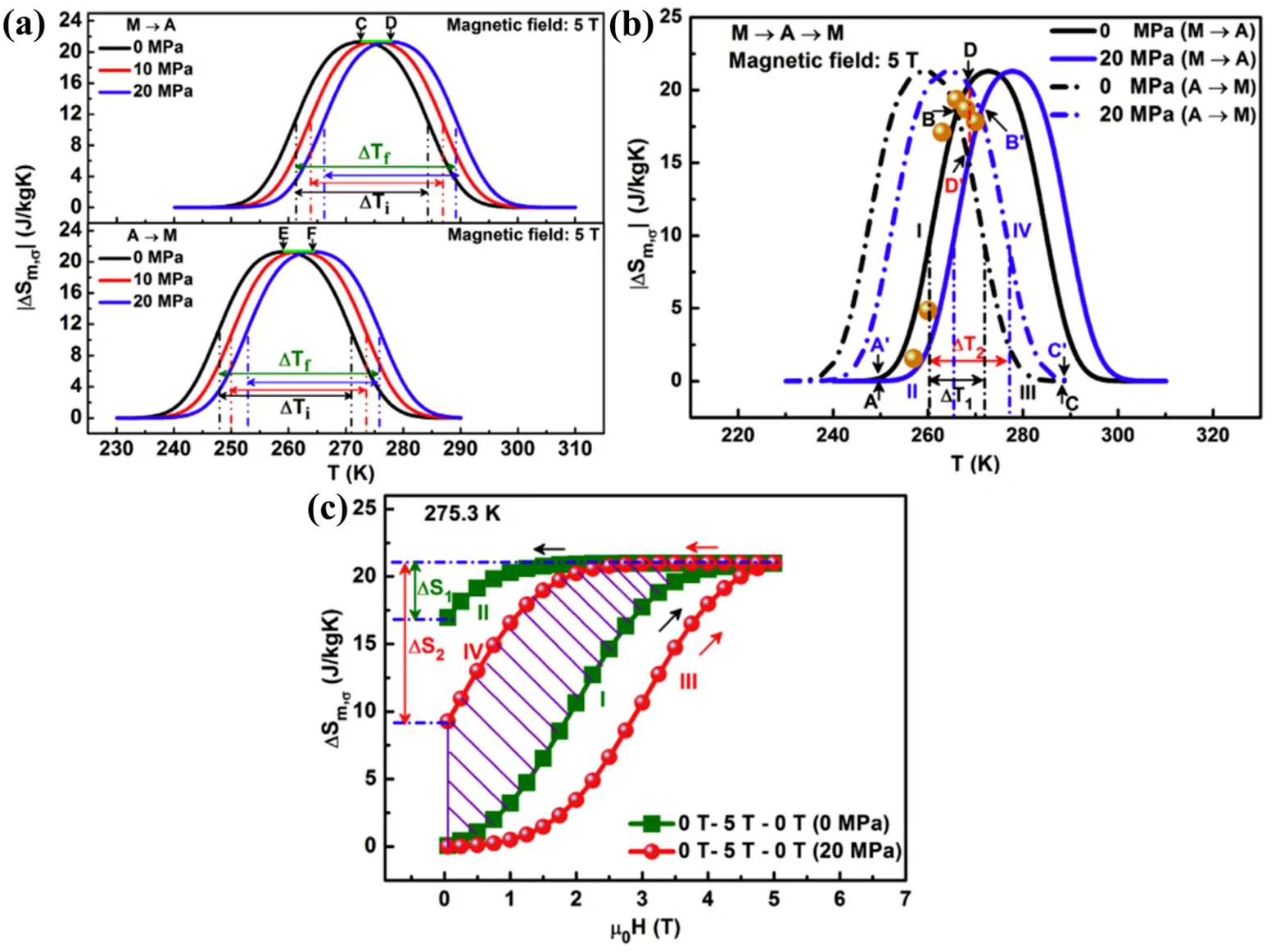
Entropy responses of Ni_43_Co_6_Mn_40_Sn_11_ alloy under coupled uniaxial stress and magnetic field: (**a**) temperature dependence of the calculated isothermal magnetic entropy change during the martensite-to-austenite (M → A) and austenite-to-martensite (A → M) transformations under a magnetic field of 5 T and stresses of 0, 10, and 20 MPa; (**b**) comparison of the M → A and A → M entropy-change curves at 0 and 20 MPa under 5 T; and (**c**) magnetic-field dependence of the entropy change during a 0–5–0 T cycle at 275.3 K under 0 and 20 MPa [5]. Copyright © 2018 Elsevier, with permission.

**Figure 3:**
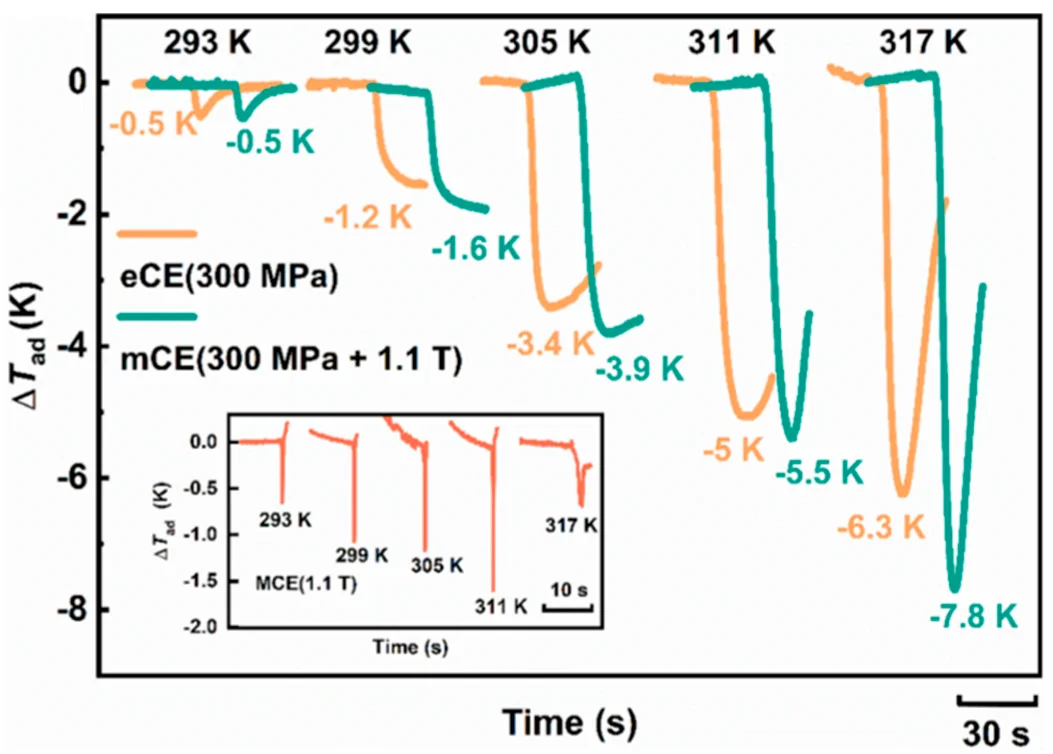
Time evolution of the Δ*T_ad_* in Ni_37_Co_13_Mn_35_Ti_15_ during unloading at 293, 299, 305, 311, and 317 K. The curves compare the elastocaloric response induced by a stress of 300 MPa (eCE) with the multicaloric response under 300 MPa and 1.1 T (mCE). The inset shows the magnetocaloric temperature change induced by 1.1 T without applied stress [33]. Copyright © 2024 Elsevier, with permission.

In Ni-Co-Mn-Sn, Ni-Fe-Co-Mn-Sn, and Ni-Co-Mn-Ti alloys, mechanical loading modifies the transformation pathway under a magnetic field, and under selected conditions, improves transformation reversibility and broadens the reversible operating-temperature range, as summarized in Figure 2 and Figure 3 [5,26,33]. The intrinsic elastocaloric response provides a useful reference for evaluating the additional contribution of magnetic–mechanical coupling. For Ni_50_Mn_31.5_In_16_Cu_2.5_, mechanical loading and unloading produced temperature changes of approximately 13 K and −10 K, respectively [34]. The unequal magnitudes indicate an asymmetric thermal response associated with transformation hysteresis and irreversible energy dissipation, as discussed further in Section 4. Phase-field simulations further reveal the evolution of microstructural and local stress fields, providing a mechanistic basis for interpreting the macroscopic caloric response [35,36].

Building on these mechanistic insights, recent theoretical and experimental studies have examined how magnetic–mechanical coupling affects transformation stress, hysteresis, reversibility, and the adiabatic temperature change (∆*T_ad_*) associated with multicaloric responses. A recent multiscale model reproduced a cooling response of 6.7 K in directionally solidified NiMnGaIn under 95 MPa uniaxial stress and a 2 T magnetic field, quantitatively linking the response to thermo-magneto-mechanical coupling and intrinsic energy dissipation [37]. In a complementary approach, Tang et al. simulated a composite Terfenol-D layer in which magnetostrictive deformation was transferred as mechanical stress to the SMA [38]. Increasing the magnetic field from 0.154 to 0.288 T increased the predicted (∆*T_ad_*) from approximately 10.8 to 12.4 K. Experimental studies provide more direct evidence of multicaloric responses under simultaneously applied uniaxial stress and magnetic field [33,39,40]. In Ni_45_Mn_44_Sn_11_, application of a 5 T magnetic field increased the critical transformation stress by approximately 20 MPa and reduced the residual strain; the measured ∆*T_ad_* reached approximately 7.4 K, nearly 20% larger than the value obtained under mechanical loading alone [39]. The practical implications of the required magnetic-field magnitude are discussed in Section 5. In Ni_37.5_Mn_35_Co_12.5_Ti_15_, simultaneous application of compressive stress and a 0.5 T magnetic field reduced mechanical hysteresis and increased the directly measured ∆*T_ad_* by approximately 36% [40]. Similarly, in Ni_37_Co_13_Mn_35_Ti_15_ at 329 K, the stress hysteresis decreased from 62.2 MPa at 0 T to 33.5 MPa at 1.1 T, corresponding to a reduction of approximately 46% [33]. This reduction is attributed to the preferential magnetic stabilization of austenite and the resulting unequal shifts in the forward and reverse transformation stresses, indicating reduced mechanical energy dissipation under the reported conditions. The underlying mechanism is discussed further within the unified framework presented in Section 6.

Comparison among these alloys shows that the benefits of stress-magnetic coupling are manifested differently across SMA systems. A moderate bias stress mainly improves transformation reversibility in Ni-Co-Mn-Sn and Ni-Fe-Co-Mn-Sn [5,26], whereas an appropriate field sequence primarily reduces stress hysteresis and energy dissipation in Ni_37_Co_13_Mn_35_Ti_15_ [33]. Ni_45_Mn_44_Sn_11_ exhibits an enhanced ∆*T_ad_*, but only under a substantially higher magnetic field [39]. These differences reflect variations in transformation strain, magnetization difference, intrinsic hysteresis, phase compatibility, texture, accessible transformation fraction, and loading sequence. Therefore, stress-magnetic coupling should be assessed in terms of reversibility, dissipation, caloric amplitude, and required field magnitude, and improvements observed under specific conditions should not be generalized to all SMA systems.

### 3.3. Hydrostatic Pressure-Magnetic Field Coupling and Multicaloric Responses

Hydrostatic pressure differs from uniaxial stress because it is nominally isotropic and couples primarily to the volume change associated with martensitic transformation. Its contribution to the phase free-energy difference can be expressed as $p{\Delta \varepsilon }_{v}^{\mathrm{tr}}$, where *p* > 0 denotes compression and ${\Delta \varepsilon }_{v}^{\mathrm{tr}}=\left(V_{M}-V_{A}\right)/V_{A}$. Pressure stabilizes the phase with the smaller volume; therefore, when martensite has a smaller volume than austenite, increasing pressure favors martensite and generally raises the transformation temperature. The direction and magnitude of this shift depend on the transformation volume and entropy changes. Unlike uniaxial stress, ideal hydrostatic pressure does not directly favor shear-related martensitic variants, although elastic anisotropy, texture, internal stress, or non-hydrostatic conditions may produce secondary variant-dependent effects.

In Ni_1.8_Pt_0.2_MnGa, pressure stabilizes martensite and alters the field-induced metamagnetic transition, while enhanced antiferromagnetic interactions reduce the magnetization difference across the transformation [28]. Similar pressure-magnetic field competition occurs in Ni-Mn-In alloys, pressure generally stabilizes martensite and raises the transformation temperature, whereas the magnetic field favors austenite and lowers it. Depending on pressure and temperature, their interaction may evolve from competitive to cooperative coupling, as illustrated by the T-P-H (T: the temperature; P: the hydrostatic pressure; H: the magnetic field) phase relations [6,25].

**Figure 4:**
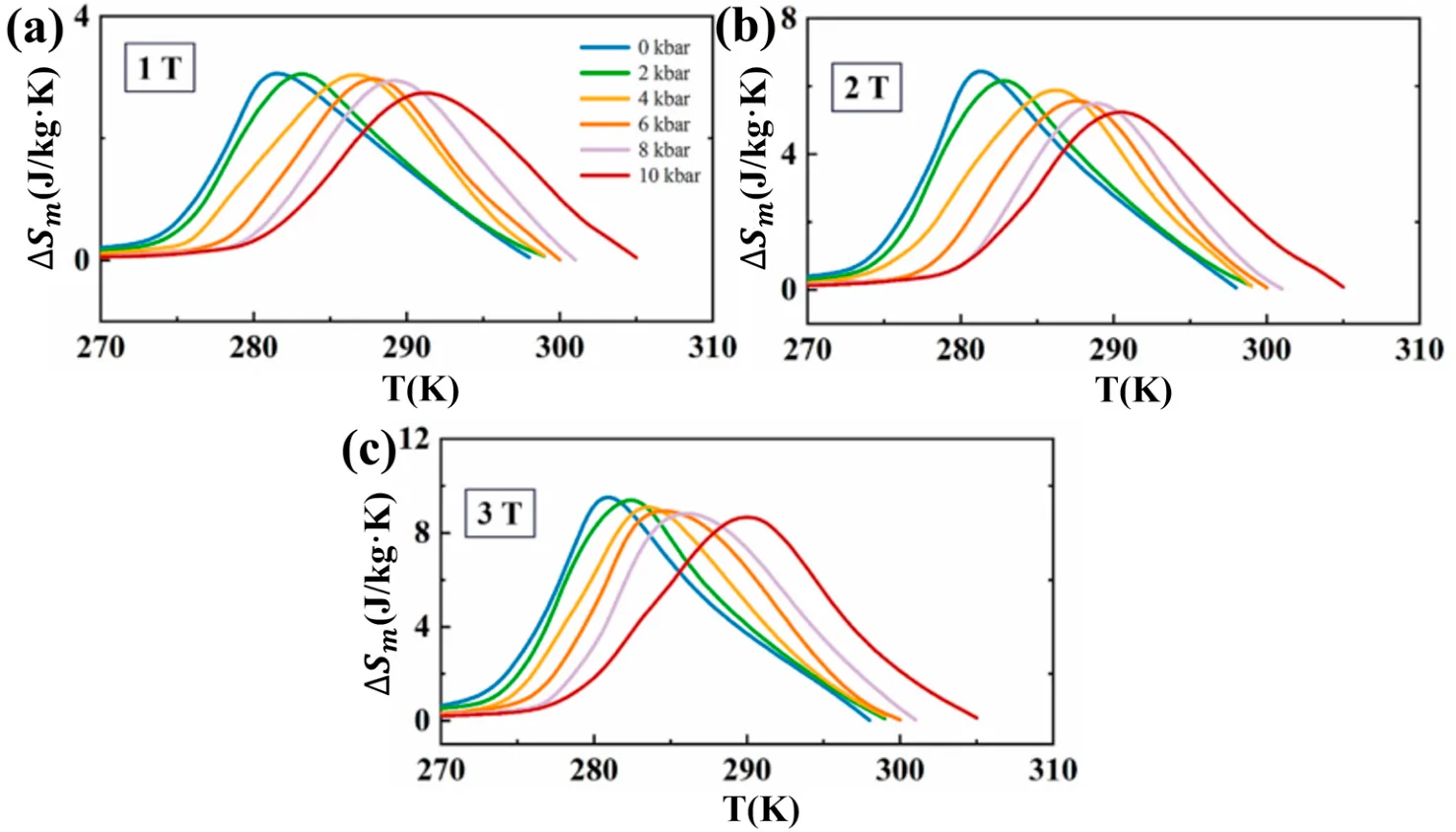
Temperature dependence of the isothermal magnetic entropy change, Δ*S_m_*, in (Ni_47_Mn_38_Sn_12_Cu_3_)_99.4_B_0.6_ under hydrostatic pressures of 0, 2, 4, 6, 8, and 10 kbar for magnetic-field changes of (**a**) 1 T, (**b**) 2 T, and (**c**) 3 T [41]. Copyright © 2025 Elsevier, with permission.

These changes in phase stability directly affect multicaloric performance. In Ni_1.8_Pt_0.2_MnGa, pressure shifts the transformation toward room temperature but reduces the magnetic entropy change (ΔS_M_) derived from the Maxwell relation [28]. This behavior indicates a trade-off between tuning the operating temperature and maintaining the magnitude of the caloric response; therefore, it should not be interpreted as evidence of improved caloric efficiency. In Ni-Mn-In alloys, pressure-magnetic field coupling produces non-monotonic entropy responses and modifies the balance between caloric magnitude and reversibility [6,25]. Similarly, in Ni-Mn-Sn-Cu-B alloys, pressure-induced shifts in transformation temperature and reduced magnetic hysteresis broaden the operating-temperature range and increase the refrigeration capacity, despite a moderate decrease in the peak magnetic entropy change, as shown in Figure 4 [41].

Comparison among these systems shows that pressure-magnetic coupling involves different performance trade-offs. Its main effect is operating-temperature tuning in Ni_1.8_Pt_0.2_MnGa [28], pressure- and temperature-dependent competition or cooperation in Ni-Mn-In [6,25], and a trade-off between peak entropy change, hysteresis, and refrigeration capacity in Ni-Mn-Sn-Cu-B [41]. These differences arise from variations in transformation volume change, magnetization difference, transformation entropy, magnetic ordering, and intrinsic hysteresis. Therefore, pressure-magnetic performance should be evaluated using reversibility and operating-temperature range together with peak Δ*S*.

Taken together, stress-magnetic and pressure-magnetic studies show that improvements in caloric amplitude, hysteresis, reversibility, and operating-temperature range do not necessarily occur simultaneously; their practical value must therefore be assessed with respect to alloy composition, field sequence, and required field magnitude.

### 3.4. Processing-Induced Influences on Subsequent Caloric Performance

Unlike the field-driven responses discussed in Section 3.2 and Section 3.3, LPBF, wire electrical discharge machining (WEDM), electric-pulse treatment, grain refinement, and compositional-gradient processing modify the initial microstructure, residual-stress state, transformation behavior, and mechanical properties of SMAs but do not act as external caloric fields during the refrigeration cycle [18]. These studies are therefore discussed separately and classified according to the reported evidence. Studies reporting only microstructural, transformation, or mechanical changes are treated as indirect evidence, whereas those measuring ΔS, ∆*T_ad_*, or exchanged heat after processing provide direct evidence of processing-modified caloric performance.

AM can tailor the microstructure and internal stress state governing martensitic transformation and subsequent caloric behavior. Laser-based fabrication modifies composition, microstructure, and residual-stress distributions in Ni-Mn-Ga alloys [42,43].

Recent LPBF process-parameter studies further show that scanning strategy and energy input regulate porosity, grain structure, texture, precipitates, internal stress, and transformation behavior in NiTi [44,45,46,47]. Magnetic-field-assisted WEDM also affects the transformation and functional behavior of NiTi [48]. However, because these studies do not directly quantify caloric parameters, they provide indirect evidence of caloric-relevant changes rather than verified improvements in caloric or multicaloric performance.

Stronger evidence is provided by studies that directly measure caloric responses after processing. Measurements on LPBF-fabricated NiTi demonstrate that the resulting microstructure affects its elastocaloric response [49], while grain-size engineering improves elastocaloric performance and cyclic stability of superelastic NiTi, and microstructural optimization influences multicaloric cooling in Ni-Mn-In alloys [50,51]. As-cast Ni_41_Fe_3_Mn_46_Sn_10_ with a dendritic microstructure exhibited an unloading ∆*T_ad_* of approximately −10.3 K at 350 MPa and retained a substantial response over 200 cycles at 250 MPa [52]. Similarly, magnetic-field-assisted directional solidification produced composition-graded Ni_50_Mn_31.5_Ti_18.5_ with a ∆*T_ad_* of approximately −18.4 K [53]. These results demonstrate that microstructures and the associated internal stress state can influence transformation reversibility and subsequently measured caloric properties, but the processing treatments should not be regarded as external caloric fields operating within the refrigeration cycle.

Electric-pulse and electric-current treatments further illustrate this distinction. In Ti-Ni alloys, electric-pulse treatment combined with deformation modifies microstructure, residual strain, and transformation stability [11,12,13], while recent work attributes improved superelasticity to the regulation of dislocations and Ni_4_Ti_3_ precipitates [54]. Because these studies did not measure ∆S, ∆*T_ad_*, or exchanged heat, they provide indirect evidence of caloric relevance. By contrast, electric-current treatment restored the elastocaloric response of functionally degraded NiTi wires [55], providing direct evidence of processing-modified caloric performance. In these studies, the electric treatment modified the material state rather than acting as an external caloric field during refrigeration.

## 4. Hysteresis, Energy Losses, and Functional Fatigue

A large caloric amplitude does not necessarily correspond to a highly reversible and recoverable cooling response, because transformation hysteresis separates the forward and reverse paths and introduces irreversible energy losses. This distinction is illustrated by Ni_50_Mn_31.5_In_16_Cu_2.5_, for which loading and unloading produced ∆*T_ad_* values of approximately +13 and −10 K, respectively, despite a thermal hysteresis of only about 3 K [34]. NiTi likewise exhibited temperature changes of approximately +30 K during loading and −19 K during unloading [56]. Across these systems, the smaller unloading response indicates that the initial caloric amplitude alone can overestimate the recoverable cooling capacity. Although irreversible energy dissipation contributes to this asymmetry, heat transfer, transformation kinetics, and incomplete phase transformation may also affect the measured values.

Coupled fields provide a route to control the balance between caloric amplitude and hysteretic loss, but their effectiveness depends strongly on the loading sequence and material system. In Ni_37_Co_13_Mn_35_Ti_15_, loading at 0 T followed by unloading under 1.1 T reduced the stress hysteresis and hysteretic energy loss by approximately 45.2% and 54.8%, respectively, while increasing the unloading |∆*T_ad_*| from 6.3 to 7.8 K [33]. In spark-plasma-sintered Ni_37.5_Mn_35_Co_12.5_Ti_15_, simultaneous mechanical and magnetic loading increased ∆*T_ad_* by approximately 36% relative to mechanical loading alone [40]. Together, these results show that coupled-field operation should be evaluated by both the enhanced caloric output and the accompanying change in hysteresis. An increase in ∆*T_ad_* alone does not demonstrate reduced dissipation unless the hysteresis or work loss is also quantified.

Cyclic results indicate that low hysteresis can favor long-term functional stability, although cyclic durability also depends on the material system, microstructure, loading mode, and field amplitude. Ni_50_Mn_31.5_In_16_Cu_2.5_, with a thermal hysteresis of approximately 3 K, maintained a stable response over 10^5^ magnetic-field cycles [34]. Under mechanical cycling, Fe-doped Ni-Mn-In retained an elastocaloric temperature change of approximately −4.5 K for more than 16,000 cycles, with no significant degradation reported under the tested conditions, whereas the corresponding single-phase alloy failed after approximately 15 cycles [57]. In contrast, during a 1000-cycle tensile test at 7% strain, the loading and unloading ∆*T_ad_* of NiTi decreased from approximately +29 and −17 K in the initial cycle to stabilized values of approximately +23 and −6 K after about 200 cycles [56]. For NiTi springs, the maximum cooling temperature retained approximately 96% of its initial value after 40 cycles at a peak displacement of 40 mm, compared with 71% at 80 mm, indicating more pronounced functional fatigue at the higher loading level [58]. These contrasting results demonstrate that fatigue resistance cannot be inferred from the initial ∆*T_ad_* and must be assessed under clearly specified cycling modes, field amplitudes, loading levels, and specimen geometries.

Overall, the available evidence indicates a practical trade-off among caloric amplitude, hysteretic loss, reversibility, and cyclic stability. Materials or loading protocols producing the largest initial ∆*T_ad_* do not necessarily provide the most durable cooling response. The practically usable response should therefore be evaluated from the stabilized loading-unloading behavior after cyclic conditioning rather than from the maximum value obtained during the initial cycle. Meaningful comparison requires simultaneous consideration of loading and unloading ∆*T_ad_*, hysteresis-loop area, energy dissipation, retained caloric response, cycle number, and failure criterion. The mechanisms connecting microstructural evolution, energy dissipation, and functional fatigue are discussed systematically in Section 6.

## 5. Quantitative Comparison and Practical Considerations

Table 1 compares representative SMA systems in terms of composition, transformation temperature, applied stress or pressure, magnetic-field strength, ∆S, ∆T_ad_, transformation stress, hysteresis, cyclic stability, and dominant caloric mechanism. Because the reported values depend on field amplitude, loading sequence, measurement protocol, and material form, these conditions should be considered when comparing different systems.

Practical performance cannot be evaluated from peak ∆*S* or ∆*T_ad_* alone. Suitable materials must combine a large reversible response with moderate stress, pressure, and magnetic-field requirements, low hysteresis, an appropriate operating-temperature range, and long cyclic life. Standardized reporting of applied-field conditions, loading and unloading responses, hysteresis, energy dissipation, and cyclic degradation is therefore required for reliable comparison and material selection.

The comparison also highlights the demanding fields used in some studies. In Ni_45_Mn_44_Sn_11_, a magnetic field of 5 T increased the critical stress by approximately 20 MPa and produced a ∆T_ad_ of about 7.4 K [39]. Although this result demonstrates strong magnetomechanical coupling, generating such a high magnetic field generally requires bulky and costly equipment, limiting its applicability in compact cooling devices. Its practical significance therefore lies primarily in demonstrating the coupling potential and motivating comparable responses under lower magnetic fields or mechanical loads.

## 6. Integrated Mechanistic Perspective of Field Coupling and Functional Fatigue

The caloric response of SMAs may be interpreted using an effective transformation driving force [27,60,61]:

(9) $$\Delta g_{\mathrm{eff}}=\Delta g_{0}\left(T\right)-\sigma \Delta \varepsilon^{\mathrm{tr}}+p\Delta \varepsilon_{v}^{\mathrm{tr}}-\mu_{0}H\Delta M+\Delta g_{\mathrm{int}}$$

Here, ∆X = X_M_ − X_A_, ∆*g_eff_* and ∆*g*_0_(*T*) denote the effective and zero-field chemical free-energy density differences, respectively; ∆M is the volumetric magnetization difference between the two phases; and ∆*g_int_* represents contributions from interfaces, defects, grain boundaries, and residual stresses. In this framework, uniaxial stress couples to the transformation strain and variant selection, hydrostatic pressure shifts the relative phase stability through the transformation volume change, and the magnetic field acts through the magnetization difference between the two phases. When applied jointly or sequentially, these fields can modify the transformed phase fraction, hysteresis, and transformation path, potentially producing a non-additive multicaloric response.

This framework explains why the stress hysteresis of Ni*_37_Co*_13_Mn*_35_Ti*_15_ decreases from 62.2 MPa at 0 T to 33.5 MPa at 1.1 T [33]. The magnetic field preferentially stabilizes austenite, increasing the stress required for forward transformation while assisting reverse transformation during unloading. The unequal shifts in transformation stresses narrow the hysteresis loop. Consequently, loading at 0 T and unloading at 1.1 T reduce stress hysteresis and energy loss by approximately 45.2% and 54.8%, respectively [33], demonstrating the importance of field sequence.

These examples illustrate distinct routes for regulating martensitic phase fraction and interface motion. Magnetic fields and Fe substitution modify phase stability in Mn_49_Ni_42−x_Fe_x_Sn_9_ [62]; texture and compositional gradients regulate the internal stress state in Ni_50_Mn_31.5_Ti_18.5_ [53]; and coupled magnetic–mechanical loading modifies the transformation response of Ni-Co-Mn-Ti [40]. Importantly, direct field-driven responses should be distinguished from processing-induced effects: the former operate during the caloric cycle, whereas the latter modify the material state before caloric measurement [18,24].

Microstructural evolution connects energy dissipation with functional fatigue. Repeated transformation generates dislocations, residual stress, retained martensite, plastic strain, and eventually cracks, which impede interface motion, increase interfacial resistance and irreversible dissipation, and reduce the reversible phase fraction and unloading ∆*T_ad_* [56,58]. Recent experiments combined with phase-field modeling show that coherent Ni_4_Ti_3_ nanoprecipitates enhance the cyclic stability of NiTi elastocaloric materials by regulating local transformation fields and suppressing irreversible microstructural evolution [59]. Other microstructural strategies, including Fe-induced dual-phase structures and directional solidification, also improve cyclic stability [53,57]. Caloric amplitude, hysteresis, and fatigue resistance must therefore be optimized together.

Current models remain limited in their ability to predict reversible and durable multicaloric responses. Equilibrium thermodynamic models describe field-dependent phase stability but generally neglect transformation kinetics, hysteresis, defect evolution, and damage accumulation. Constitutive models can reproduce macroscopic stress–strain or caloric responses but often rely on phenomenological parameters that are not readily transferable among different compositions, microstructures, and loading paths. Phase-field models provide valuable microstructural insight but often rely on literature-derived interfacial, kinetic, and elastic parameters and remain limited in accessible spatial and temporal scales. For example, the recent NiTi model employed a two-dimensional single-crystal representation and could not reproduce high-cycle deformation because of computational limitations [59]. Moreover, the effects of stress, pressure, and magnetic field are frequently modeled or calibrated separately, potentially overlooking non-additive coupling and field-sequence effects.

Establishing a unified quantitative framework therefore requires standardized measurements on the same specimen under controlled single-, combined-, and sequential-field protocols. Essential experimental data include field magnitude, application rate and sequence, transformation temperatures, transformation strain and volume change, magnetization difference, entropy change, heat capacity, phase fraction, transformation rate, loading and unloading ∆*T_ad_*, hysteresis-loop area, residual strain, retained caloric response, cycle number, and failure criterion. Required model parameters include nucleation barriers, interfacial energy and mobility, elastic constants, internal friction, defect-generation and evolution laws, heat-transfer coefficients, and damage-accumulation parameters. Integrating these quantities within a coupled thermodynamic–kinetic–damage framework is necessary to predict reversible and long-term stable cooling under device-relevant fields.

## 7. Conclusions

This review clarifies the distinct roles of uniaxial stress and hydrostatic pressure in the multicaloric behavior of shape memory alloys. Uniaxial stress acts through transformation strain and variant selection, whereas hydrostatic pressure modifies phase stability through the transformation volume change. Their coupling with magnetic fields can alter the phase fraction, transformation path, hysteresis, and caloric response. These interactions may be cooperative or competitive and depend on alloy composition, microstructure, temperature, field magnitude, and loading sequence.

Representative results demonstrate the potential of coupled-field protocols under specific material and loading conditions. In Ni_37_Co_13_Mn_35_Ti_15_, loading at 0 T followed by unloading at 1.1 T reduced stress hysteresis and hysteretic energy loss by up to 45.2% and 54.8%, respectively, while increasing the unloading |∆*T_ad_*| from 6.3 to 7.8 K [33]. Simultaneous mechanical and magnetic loading produced a reported increase in ∆*T_ad_* of approximately 36% in Ni_37.5_Mn_35_Co_12.5_Ti_15_ [40]. However, the contrasting cyclic responses of different SMAs show that reduced hysteresis and improved stability cannot be generalized. Caloric amplitude, reversibility, energy dissipation, field magnitude, and functional fatigue must therefore be evaluated together.

Direct field-driven responses should also be distinguished from processing-induced changes that indirectly affect caloric behavior. The development of a unified quantitative framework is currently hindered by the limited availability of standardized same-specimen measurements under controlled field sequences. Required data include ∆*T_ad_*, ∆*S*, phase fraction, transformation kinetics, hysteresis loss, and cycle-dependent degradation, together with parameters describing transformation strain, volume change, magnetization difference, interface mobility, kinetic barriers, and damage evolution. Integrating these quantities into a coupled thermodynamic–kinetic–damage framework would provide a basis for predicting reversible and durable multicaloric cooling under device-relevant fields.

## Figures and Tables

**Table 1:** Quantitative comparison of representative caloric and multicaloric responses in selected SMA systems.

Alloy	Loading Mode	*T_tr_* (K)	*σ* (MPa)	*P* (GPa)	*μ*_0_*H* (T)	Δ*S* (J kg^−1^K^−1^)	∆*T_ad_* (*K*)	Transformation Stress/Other Indicator	Hysteresis/ Loss	Cyclic Stability	Dominant Caloric Mechanism	Ref.
Ni_43_Co_6_Mn_40_Sn_11_	*σ* + *H*	Near RT	NR	_	5	19.3	7.7, estimated MCE; 7.1, direct eCE	Stress-assisted reversible transformation	Hysteresis width NR	Stable over 380 thermomagnetic cycles	Magnetization-transformation-strain coupling	[5]
Ni-Mn-In	*P* + *H*	NR; pressure increases *T_tr_*	_	NR	NR	NR; non-monotonic pressure dependence	NR	Pressure- and field-dependent phase stability	NR	NR	Volume-change-magnetization coupling	[6]
Ni_42.5_Fe_1.0_Co_6.5_Mn_39.5_Sn_10.5_	*σ* + *H*	Near RT	Low bias stress; value NR	_	≤6	NR	NR	Improved transformation reversibility	NR	NR	Magnetization-transformation-strain coupling	[26]
Ni_1.8_Pt_0.2_MnGa	*P* + *H*	*dT_tr_*/*dp* ≈ 3.5 KGPa^−1^	_	*P* = 0–0.91	5	9.31 → 5.52	NR	Pressure stabilizes martensite and shifts *T_tr_*	Magnetic hysteresis reduced; value NR	NR	Pressure-controlled phase stability and MCE	[28]
Ni_54_Mn_23_Ga_23_	*P*	*T_M_*/*T_A_* ≈ 300/315	_	0.74	_	16.86, heating; 16.73, cooling	9.3, calculated	*dT_M_*/*dp* = 15.89 K GPa^−1^	Thermal hysteresis ≈15 K; nearly pressure-independent	NR	Volume-change-driven BCE	[29]
Ni_26_Cu_22_Mn_46_Sn_6_	*P*	NR; peak Δ*S* at ≈242.8 K	_	0.10	_	−40.47, quasi-direct	18.23, estimated	*dT*/*dp* = 3.90/2.35 K kbar^−1^, cooling/heating	Thermal hysteresis: 5.3 K at 1 kbar	NR	Composition-designed, volume-change-driven BCE	[30]
Ni_37_Co_13_Mn_35_Ti_15_	*σ* + *H*	*M_s_*/*M_f_* = 289.9/276.4; *A_s_*/*A_f_* = 291.0/306.1	300	_	1.1	22.5, heating; 19.4, cooling	−6.3 *to* −7.8	Loading at 0 T and unloading at 1.1 T	Stress hysteresis: 62.2 → 33.5 MPa; energy loss reduced by 54.8%	NR	Magnetization-transformation-strain coupling	[33]
Ni_50_Mn_31.5_In_16_Cu_2.5_	*σ*; magnetic cycling	Near RT	NR	_	2	NR	+13/−10	Asymmetric loading-unloading response	Thermal hysteresis: ≈3 K	Stable over 10^5^ magnetic-field cycles	Stress- and magnetic-field-driven transformation	[34]
LPBF Ni_45_Mn_44_Sn_11_	*σ* + *H*	Near RT	NR	_	5	NR	≈7.4	Critical transformation stress increased by ≈20 MPa; residual strain reduced	NR	NR	Magnetization-transformation-strain coupling	[39]
SPS Ni_37.5_Mn_35_Co_12.5_Ti_15_	*σ* + *H*	*A_s_*/*A_f_* = 301/319; *M_s_*/*M_f_* = 305/280	550	_	≤5	19–22 at 5 T	≈3.7	Magnetic field increases the caloric response	Thermal hysteresis: 14 K; Mechanical hysteresis reduced under 0.5 T; value NR	NR	Magnetization-transformation-strain coupling	[40]
(Ni_47_Mn_38_Sn_12_Cu_3_)_99.4_B_0.6_	*σ*, *P*, *H*	Near RT	300–500	NR	5	21.2; ∆S_tr_ ≈ 20.6	5.1 to 10.3	Pressure shifts the transformation temperature	Magnetic hysteresis reduced by pressure; value NR	NR	Stress/pressure-magnetic coupling	[41]
LPBF NiTi	Compression, after LPBF	Sample-dependent; tested at *A_f_* + 10 *K*	NR; 6% strain	_	_	NR	Cooling: 11.0 → 9.9 → 8.7 K, cycles 1 → 5 → 10	Recoverable strain up to 80.17% after 10 cycles	Stress hysteresis composition-dependent; value NR	10 compression cycles	Processing-modified eCE	[49]
Grain-engineered NiTi	*σ*	NR; grain-size dependent	NR	_	_	NR	Value NR in this comparison	Grain-size-dependent transformation behavior	NR	Improved; cycle number NR	Microstructure-controlled eCE	[50]
Ni_41_Fe_3_Mn_46_Sn_10_	*σ*	Tested at 300 K	200–350	_	_	NR	−4.4 to −10.3	Residual strain ≈ 0.5%	NR	<1 K loss after 200 cycles at 250 MPa	Microstructure-controlled eCE	[52]
Ni_50_Mn_31.5_Ti_18.5_	*σ*, after directional solidification	263–463	≈390 at 293 K	_	_	NR	max. −18.4	Texture- and composition-gradient-controlled transformation	Stress hysteresis: ≈63 MPa	−7.0 → −6.5 K after mechanical 500 cycles	Internal-stress- and texture-mediated eCE	[53]
Ti_49_Ni_51_ wire, after current treatment	Tensile, 3% strain	*A_f_* ≈ 302 K initially	NR	_	_	NR	NR; initial measured temperature rise 11.2 ± 0.4 K	Temperature rise/drop recovered by ≈14%/6%	NR	1000 cycles; treatment every 100 cycles	Current-treatment recovery of eCE	[55]
NiTi	Tensile *σ*	*A_f_* ≈ 283	≈420–480; 7% strain	_	_	≈35.1	+30/−19	Loading-unloading thermal asymmetry	Frictional/hysteretic loss; value NR	+29/−17 → +23/−6 K over 1000 tensile cycles	eCE	[56]
Fe-doped Ni-Mn-In (Fe6)	*σ*, *H*	*M_s_*/*M_f_* = 288.8/283.6; *A_s_*/*A_f_* = 294.2/299.1	300	_	≈2	−21.1/22.8, DSC; ≈15, magnetic	eCE ≈ −4.5; MCE ≈ −3	Dual-phase microstructure improves functional stability	Thermal hysteresis 10.5 K	>16,000 mechanical cycles	Stress- and magnetic-field-driven transformation	[57]
NiTi springs	Displacement controlled	*A_f_* ≈ 284	40–80 mm displacement	_	_	NR	Cooling: 5.5 to 13.6	Cooling response increases with displacement	NR	96% and 71% retained after 40 cycles at 40 and 80 mm, respectively	Component- level eCE	[58]
Ni_50.8_Ti_49.2_ with Ni_4_Ti_3_ nanoprecipitates	Tensile	Tested at ≈300 K	600	_	_	NR	10.5	*COP_mat_* ≈ 46.5; ≈15 nm precipitates	Energy dissipation stabilized; value NR	Stable over 100 cycles	Precipitate-stabilized eCE	[59]

Notes: *T_tr_*, transformation temperature; RT, room temperature; BCE, barocaloric effect; MCE, magnetocaloric effect; eCE, elastocaloric effect; SPS, spark-plasma sintering; NR, not reported; _, not applicable. Thermal, stress, and magnetic hysteresis and dissipated energy are distinct quantities; their types and units are therefore specified separately. Directly measured, calculated, estimated, and quasi-direct caloric values are identified because results obtained using different field histories and measurement protocols are not directly comparable. For current-treated TiNi wire, the reported temperature change was not explicitly identified as ΔT_ad_. Fields used only during processing are excluded from the operating-field columns because processing treatments are not considered external caloric fields. The dominant caloric mechanisms are listed for comparison, while their detailed thermodynamic and microstructural origins are discussed in Section 6.

## Data Availability

No new data were created or analyzed in this study. Data sharing is not applicable to this article.
